# A Comparison of Two Delivery Modalities of a Mobile Phone-Based Assessment for Serious Mental Illness: Native Smartphone Application vs Text-Messaging Only Implementations

**DOI:** 10.2196/jmir.2328

**Published:** 2013-04-05

**Authors:** John Ainsworth, Jasper E Palmier-Claus, Matthew Machin, Christine Barrowclough, Graham Dunn, Anne Rogers, Iain Buchan, Emma Barkus, Shitij Kapur, Til Wykes, Richard S Hopkins, Shôn Lewis

**Affiliations:** ^1^NIBHI Manchester Health e-Research CentreInstitue of Population HealthUniversity of ManchesterManchesterUnited Kingdom; ^2^School of Psychological ScienceDivision of Clinicial PsychologyUniversity of ManchesterManchesterUnited Kingdom; ^3^Centre for BiostatisticsInstitute of Population HealthUniversity of ManchesterManchesterUnited Kingdom; ^4^Health SciencesUniversity of SouthamptonSouthamptonUnited Kingdom; ^5^NIBHI Manchester Health e-Research CentreInstitute of Population HealthUniversity of ManchesterManchesterUnited Kingdom; ^6^School of PsychologyUniversity of WollongongWollongongAustralia; ^7^Institute of PsychiatryKings's College LondonLondonUnited Kingdom; ^8^Manchester Mental Health and Social Care TrustManchesterUnited Kingdom; ^9^Institute of Brain, Behaviour and Mental HealthUniversity of ManchesterManchesterUnited Kingdom

**Keywords:** mobile phone, psychosis, assessment, schizophrenia, text-messages

## Abstract

**Background:**

Mobile phone–based assessment may represent a cost-effective and clinically effective method of monitoring psychotic symptoms in real-time. There are several software options, including the use of native smartphone applications and text messages (short message service, SMS). Little is known about the strengths and limitations of these two approaches in monitoring symptoms in individuals with serious mental illness.

**Objective:**

The objective of this study was to compare two different delivery modalities of the same diagnostic assessment for individuals with non-affective psychosis—a native smartphone application employing a graphical, touch user interface against an SMS text-only implementation. The overall hypothesis of the study was that patient participants with sewrious mental illness would find both delivery modalities feasible and acceptable to use, measured by the quantitative post-assessment feedback questionnaire scores, the number of data points completed, and the time taken to complete the assessment. It was also predicted that a native smartphone application would (1) yield a greater number of data points, (2) take less time, and (3) be more positively appraised by patient participant users than the text-based system.

**Methods:**

A randomized repeated measures crossover design was employed. Participants with currently treated Diagnostic and Statistical Manual (Fourth Edition) schizophrenia or related disorders (n=24) were randomly allocated to completing 6 days of assessment (four sets of questions per day) with a native smartphone application or the SMS text-only implementation. There was then a 1-week break before completing a further 6 days with the alternative delivery modality. Quantitative feedback questionnaires were administered at the end of each period of sampling.

**Results:**

A greater proportion of data points were completed with the native smartphone application in comparison to the SMS text-only implementation (β = -.25, SE=.11, *P*=.02), which also took significantly less time to complete (β =.78, SE= .09, *P*<.001). Although there were no significant differences in participants’ quantitative feedback for the two delivery modalities, most participants reported preferring the native smartphone application (67%; n=16) and found it easier to use (71%; n=16). 33% of participants reported that they would be willing to complete mobile phone assessment for 5 weeks or longer.

**Conclusions:**

Native smartphone applications and SMS text are both valuable methods of delivering real-time assessment in individuals with schizophrenia. However, a more streamlined graphical user interface may lead to better compliance and shorter entry times. Further research is needed to test the efficacy of this technology within clinical services, to assess validity over longer periods of time and when delivered on patients’ own phones.

## Introduction

Schizophrenia is a major public health problem affecting approximately 1% of the general population. It represents both a substantive emotional burden for those involved and a socioeconomic burden in the United Kingdom [1]. There is now increasing emphasis on treating patients within the community [2]. However, symptoms, mood, and functioning can change suddenly, potentially leading to relapse, unscheduled acute care, or self-injury. Monitoring of psychotic symptoms usually relies on interview assessments infrequently conducted by clinical staff, with limited resources, reducing the capacity to detect sudden change. Additionally, interviews relying on retrospective accounts introduces bias and averaging, thereby losing clinical information [3]. A more sensitive approach would be to closely monitor patients during the course of their everyday lives. Real-time assessment of psychotic symptoms is attractive in that it can lead to early and immediate intervention, potentially preventing the deterioration of an individual’s mental state.

Over the past decade, assessment technologies have been increasingly employed in the monitoring of psychosis [4]. Typically, patients complete self-report questions at several different times of the day in real-world settings [5]. Early research in this field focused on the use of Personal Digital Assistants (PDAs) [6,7], but these technologies are becoming increasingly obsolescent and now occupy a very small share of the commercial market. Mobile phones have the advantage that they allow the automatic and wireless uploading of information to a central computer. As mobile phone technology becomes increasingly widespread, software applications can operate from users’ own phones, omitting the need for them to carry an additional device. In a recent study, we observed that 83% (n=30/36) of a sample with psychosis currently owned a mobile phone, suggesting that usage in individuals with psychosis is comparable to the general population in the United Kingdom. Other studies have also reported high levels of familiarity with and access to mobile phone technology in individuals with serious mental illness [8].

There are multiple ways in which mobile phone-based assessment can be employed in health care. Text messages have the advantage that they are relatively easy and inexpensive to deliver and are not limited by the model or make of an individual’s phone [9]. Individuals will likely be familiar with sending and receiving text messages. To date, two studies have used text messaging in the clinical care of psychotic illness. Španiel and colleagues [10] used weekly text-based assessments in order to alert consulting psychiatrists to early signs of relapse in patients with psychosis, which significantly reduced the number of inpatient admissions over one year. Additionally, Granholm and colleagues [11] have used text messages to promote cognitive behavioral therapy techniques and health-promoting behaviors. Pilot data showed a significant increase in social interaction and a reduction in hallucinations, but no significant effect on medication use.

Native software applications, with graphical user interfaces, uploaded onto smartphone devices pose another viable option for conducting real-time assessment. For the purpose of this research, a smartphone will be defined as having (1) computing power, (2) a touch screen, (3) third party application development and distribution, and (4) the option of 3G connectivity and high-speed data transfer. Native smartphone applications can be purpose built allowing for greater flexibility and ease of use [9] and are now increasingly being used in the treatment of physical health problems (eg, diabetes) [12]. Our research group has recently developed one such software application for use on Android smartphones. This software enables users to respond to self-report questions relating to their symptoms on touch-screen analogue scales. Initial piloting of this technology showed little dropout and high levels of data points completed in 44 individuals experiencing psychosis, sampled for 1 week. 13 self-assessment scales representing different dimensions of psychotic illness were validated against commonly employed, gold-standard clinical interviews. However, further information is required to assess the benefits and limitations of native smartphone applications in comparison to other methods of mobile phone-based data collection.

The objective of this study was to compare two different delivery modalities of the same diagnostic assessment for individuals with nonaffective psychosis—a native smartphone application employing a graphical, touch user interface against an short message service (SMS) text-only implementation. It was thought that both approaches would have certain advantages in the real-time assessment of psychosis but that the native smartphone application would display greater usability and functionality. Understanding the appropriate technology, user interface, procedures, and methods for administering mobile phone–based assessment is an important step in making it both cost- and clinically effective, with the eventual aim of integrating it into long-term illness management.

The overall hypothesis of the study was that patient participants with serious mental illness would find both the smartphone application and SMS text-only implementation feasible and acceptable to use, measured by quantitative postassessment feedback questionnaire scores, the number of data points completed, and the time taken to complete the assessment. We also had three more specific predictions: (1) that the participants would complete a significantly greater number of entries on a native smartphone application when compared to a SMS text-only implementation, (2) that entries would take significantly longer to complete on a SMS text-only implementation, and (3) that the smartphone application would be appraised more favorably in quantitative feedback scores. We also asked participants about the maximum length of time that individuals thought they would be willing to complete assessment over for each delivery modality.

## Methods

### Participants

24 community-based patients meeting or having met the criteria for a Diagnostic and Statistical Manual (Fourth Edition) diagnosis of schizophrenia (n=22) or schizoaffective disorder (n=2) as made by the clinical service and checked against DSM-IV criteria by the researcher were admitted to the study. All participants were aged 18-50 and provided informed consent to take part. Participants were required to own and have access to a mobile phone with which to use the SMS text-only implementation. Organic or substance induced psychosis were exclusion criteria.

The sample was predominantly male (79%; n=19) and white British (71%; n=17) with a mean age of 33.0 (SD 9.5, min=18, max=49). Patients were recruited through Community Mental Health Teams (63%; n=15), Early Intervention Services (33%; n=8) and through a supported-living organization (4%; n=1). The mean number of past hospital admissions in the sample was 2.3 (SD 2.5, min = 0, max = 9) and the majority of the sample were currently taking antipsychotic medication (n=20). The Positive and Negative Syndrome Scale (PANSS [13]) is taken as a gold standard-clinical measure commonly used to assess psychosis. PANSS scores in this sample (at baseline) varied considerably between individuals, suggesting a range of symptom severities (positive subscale: mean 15.0 (SD 4.5, min = 7, max = 28); negative subscale: mean 12.3 (SD 3.5, min = 7, max = 21); general subscale: mean 28.8 (SD 6.8, min = 18, max = 50).

### Equipment

The two different modalities for delivering real-time assessment, namely a smartphone application and an SMS text-only implementation, were designed such that protocol for the monitoring procedure was common to each. The core functionality for the protocol that is common to both modalities is shown in Table 1.

From the end user’s perspective, the two modalities differ only in the way the participant interacts with the device. These differences are summarized in Table 2.

**Figure 1:**
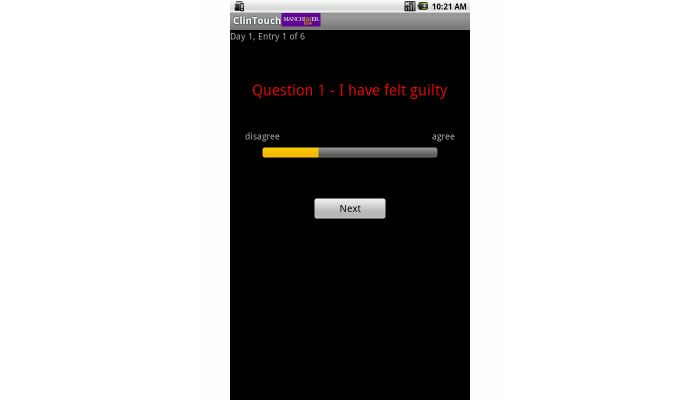
A typical question from the Android app implementation, showing the full screen with the question and the slider for data entry.

**Figure 2:**
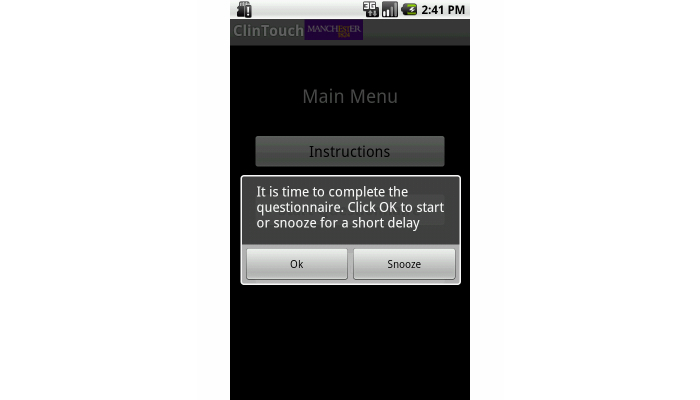
The start screen shown to the user in the Android app at the start of each set of questions (from which the users may proceed or delay ["snooze"] for a further 10 minutes they wish).

The native smartphone application was specifically developed for Android mobile phones (Figures 1 and 2). Android is an operating system created by Google, which runs on mobile phones from different manufacturers. Although this software was developed to run on any Android phone, for this study we used the Orange San Francisco device. For the purpose of this study, it was not wirelessly enabled and all answers were stored on the mobile phone handset for downloading at the end of the sampling procedure. However, this made no difference to the user’s perspective of the software.

**Figure 3:**
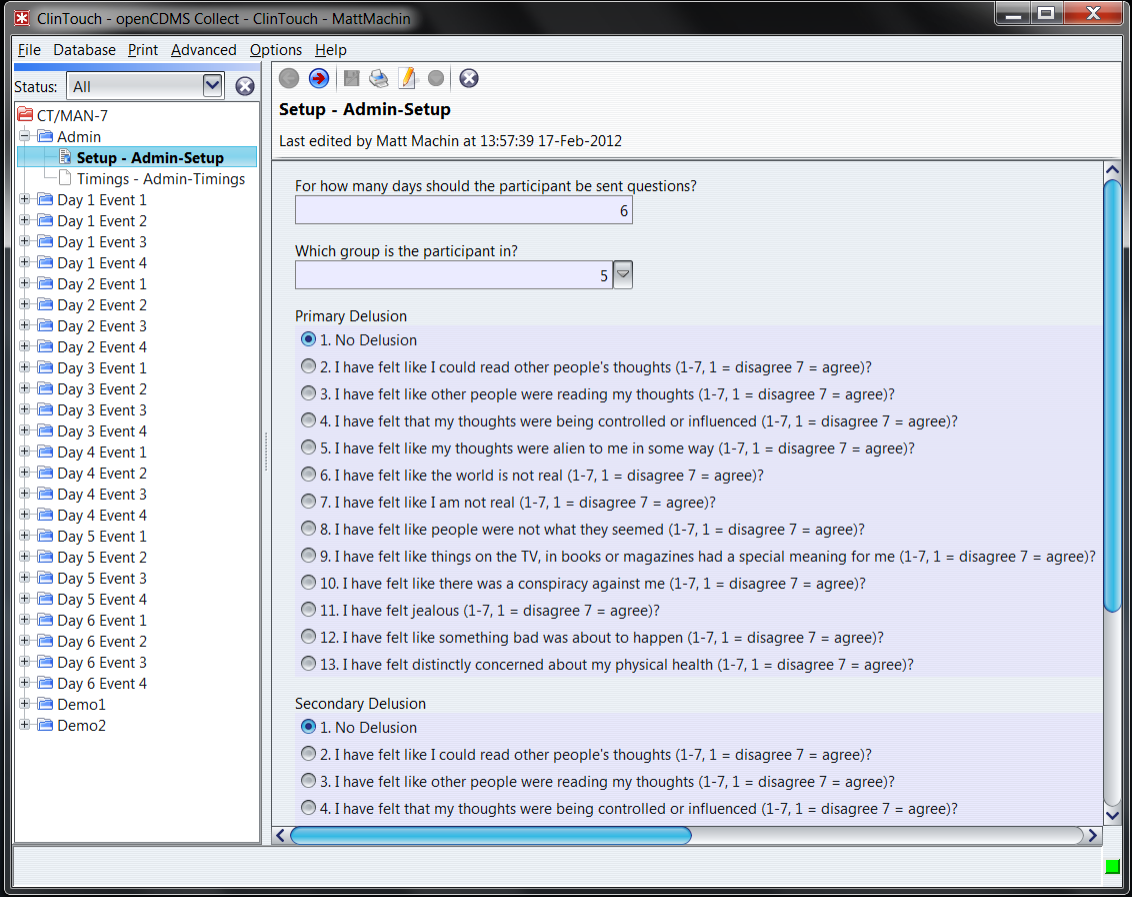
SMS configuration screen implemented in openCDMS.

The SMS text-only implementation was driven by openCDMS [14], an open source, secure online platform, which facilitated both the sending of questions and the storing of responses (Figure 3). openCDMS is a Web-enabled server-side application written in Java developed for electronic data collection in clinical studies and trials. It had pre-existing capabilities for SMS text messaging, individual participant records, and study questionnaires. Where new functionality was needed for the text message system, it was specifically developed.

### Semistructured Interviews

The PANSS [13] was conducted by an experienced administrator before and after each period of sampling. The PANSS is a semistructured interview assessing positive (7 items), negative (7 items), and global (16 items) symptoms of psychosis and has been extensively validated [13,15]. In this study, the PANSS was employed to provide convergent validity to the adapted mobile phone assessment depression items and to determine which form of delusions should be assessed.

### Quantitative Feedback Questionnaire

A purpose-designed quantitative feedback questionnaire was developed to assess the acceptability and feasibility of the native smartphone application and SMS text-only implementations (see Tables 3 and 4). This included reactivity to the methodology and whether it had been successfully integrated into an individual’s everyday routine. Three items were taken from a previous study assessing the use of PDAs in individuals with psychosis [16]. These were “Overall, this was stressful”, “Overall, this was challenging”, and “Overall, this was pleasing”. All items were rated from 1 (Not at all) to 7 (Very much so). In order to gauge the feasibility of the two methods of mobile phone assessment in the long-term management of patient’s symptoms, participants were also asked to the maximum length of time within which they hypothetically would be willing to complete questions with each delivery modality. At the end of the study, participants were also asked which delivery modality they found easier to use and which they preferred (smartphone application, SMS text-only implementation, or no preference).

**Table 1:** Core functionality common to both the native smartphone application and text-message systems.

Configurable number and times of question sets each day	At the start of the study, this can be configured for the desired number of questions sets and the times of these questions. During this study, 4 question sets per day were used.
Configurable questions	The wording of the questions is set up at the start of the study. It is easily configurable to support multiple studies with different questions. In addition, delusion questions are configured at the point when the researcher meets with the participant, through an administrative dialogue.
Multiple question sets	Multiple sets of questions are supported, and the software will switch between these at each consecutive alarm. So, if there are 2 question sets, it will ask set 1 at the first alarm, set 2 at the second, set 1 at the third, etc. The only exception to this is if the participant fails to answer some of the questions. In that case, the same question set is presented again at the next alarm.
Question branching	The next question displayed to the user can depend on the answer to one or more previous questions. This allows the questions asked to match the participant’s symptoms or situation. For example, if a participant does not endorse the first question about a particular psychotic symptom, all remaining questions about it will be skipped. This means that the participant does not waste time answering unnecessary questions.
Questionnaire timeout	A time window is enforced, within which the questionnaire has to be completed. No further answers will be accepted outside of this time window.
Logging	The time at which each question was answered is recorded to the nearest second. This can be used to analyze the time taken to answer each question as well as the time to complete the whole questionnaire.
Branching logic	A range of different branching logic types is available. In the following list, the first three items apply to branching based on an answer to a single question. All other items are applicable when branching is based on answers to multiple questions. Less than Greater than Equal to Greater than sum Less than sum One is less One is greater All are less All are greater

**Table 2:** Human-machine interface difference between native smartphone application and SMS text-only implementations of the common diagnostic assessment.

	Native smartphone application	SMS text message
Alerts	Reuses Android’s Alarm Manager so user definable alerts are available; delivered at semirandom intervals during the data collection period; users can snooze the alert to be reminded 5 minutes later. Only a single alert for each question set.	Alert is the phone’s SMS alert, triggered when a SMS question is received; delivered at semirandom intervals during the data collection period. As each question is delivered as an SMS text message, an alert is triggered for each question. A reminder SMS is sent after 5 minutes if no response is received.
Questions	Presented as one question per page in the application. User is able to navigate through the pages of questions.	Delivered as SMS text messages one question per SMS. User must respond with an SMS text message and wait for the next question in the set.
Data input	Continuous slider bar, user slides with finger touching screen. Position of slider mapped to 7-point Likert scale.	User enters number between 1 and 7 as a response.
Saving data	Fully automatic, no user input required	User must send SMS text message containing the response value in reply to the question to record their response.

### Diagnostic Assessment Items

Seven different symptom dimensions were assessed using previously validated mobile phone assessment scales. In order to minimize the burden on participants, these were split into two alternating sets. In set one, hopelessness (2-4 items), depression (2-6 items), and hallucinations (2-8 items) were assessed; and in set two, anxiety (1-4 items), grandiosity (2-3 items), paranoia (3-6 items), and delusions (0-8 items) were examined. Therefore, although there were four assessment points, each set of questions was assessed only twice per day. All questions related to the period of time that had elapsed since the last entry. Items were branched so that the questions changed depending on an individual’s previous responses.

The depression subscale was adapted from the original validation study in an effort to increase its association with the PANSS depression scale, since the correlation was moderately low. The new subscale consisted of the items: “I have felt miserable” (new), “I have had no interest in seeing other people” (new), “I have felt worthless” (new), “I have felt sad”, “My mood has affected my appetite or sleep”, and “I have had thoughts about harming myself”. As a result, the correlation between the mobile phone assessment scale and the PANSS was increased from rho = 0.45 (*P*=.01) in the original research to rho = 0.56 (*P*=.01) in the current study (calculated for the week of sampling with the native smartphone application). A strong correlation indicates that the interview and mobile phone–based assessments are tapping into similar concepts. The adapted depression assessment scale also showed considerable variability across time (mean squared successive difference: 2.2 (SD 2.9); within participant standard deviation: 0.8 (SD 0.6), suggesting that it was sensitive to subtle fluctuations in mood.

A wide range of delusions have been reported in the psychosis literature [17,18]. Therefore, during the initial briefing session, the researcher selected which delusion items were presented to the participant through the administrative dialogue. The choice of items was based on consultation with clinical staff and an initial PANSS interview to assess symptoms. For those individuals experiencing three or greater delusions, those two with the greatest conviction and distress were entered. The frequency of the different delusions were: “I have felt like other people were reading my thoughts” (n=5), “I have felt that my thoughts were being controlled or influenced” (n=4), “I have felt like I could read other people’s thoughts” (n=3), “I have felt like things on the TV, in books or magazines had a special meaning for me” (n=3), “I have felt like something bad was about to happen” (n=2), “I have felt distinctly concerned about my physical health” (n=2), and “I have felt like my thoughts were alien to me in some way” (n=1). The delusion items were kept constant for each participant across the two conditions of the trial.

### Procedure

A randomized repeated measures crossover design was employed. Participants were randomly allocated to either completing 6 days of sampling using the native smartphone application implementation on a smartphone provided to them for the purpose of the study, or via SMS text-only implementation using their own phone. There was then a 7-day rest period in order to reduce carryover effects before the individual completed a further 6 days of sampling with the alternate delivery modality. Each participant, therefore, completed two periods of sampling: 1 week with the native smartphone application implementation and 1 week with the SMS text-only implementation. Randomization was achieved through the openCDMS software. openCDMS uses a permuted block randomizer, and in this case we used a minimum block size of 4 and maximum of 6.

The researcher initially met with participants to obtain written consent and demographic information, complete clinical interviews, provide training in the first delivery modality, and administer practice questions. The participant number and delusion items were set by the researcher on either a password-protected administrators’ page (on the native smartphone application) or through the openCMDS website (on the SMS text-only implementation). The volume of the alarm prompts was also set according to the preference of the participant when using the native smartphone application implementation.

On each day of sampling, participants could complete a maximum of four sets of questions, available at pseudorandom times (selected by a random number generator at least 1 hour apart) when prompted by the mobile phone device between 09:00 and 21:00 hours. All participants had 15 minutes from the first alarm within which to complete the questions. A forced entry time was thought to prevent a self-selection bias (ie, answering the questions only when asymptomatic) [19]. The researcher telephoned once or twice (as per the participant’s preference) during the week in order to encourage compliance, answer questions, and to ascertain any problems with the software. The researcher attempted to keep the number of calls made to participants balanced over the two conditions: smartphone: mean, 1.7 (SD 0.5); text-messages: mean, 1.7 (SD 0.6).

Upon completion of the first week of sampling, the researcher met with the participant to re-administer the clinical interviews and to gather quantitative feedback on the first device. This procedure was then repeated the following week with the alternate device. At the end of the study, participants rated which device they preferred and found easier to use. Qualitative interviews were also conducted (the results of which are provided in a separate manuscript).

Participants on pay-as-you-go tariffs received £50 worth of phone credit, which they topped up prior to the week of text-message questions. If the participant was on a contract tariff (ie, direct debit) but did not have unlimited free texts, then they were reimbursed £50 at the end of the study. All participants were reimbursed an additional £30 upon completion of both sampling procedures.

### Statistics

All analysis was conducted in Stata 10.0 [20]. First, it was important to determine whether there was an interaction between period and delivery modality allocation. Delivery modality order (smartphone application then SMS text-only implementation or SMS text-only implementation then smartphone application) was entered into regression analysis as a predictor of the total score for each of the three outcome measures (mean time taken for each entry, number of completed data points, or quantitative feedback score) summed across the two conditions. A Spearman correlation was used to assess the similarity between scores across the two conditions.

Subsequent analysis was performed in a nested (long) form of the data. This data structure violates the assumption of independent observations meaning that additional constraints need to be placed on the statistical models. Multilevel modelling (xtreg) was therefore used to investigate whether delivery modality (smartphone application or SMS text-only implementation) or time-point (week 1 or week 2) significantly predicted any of the outcome variables. The outcomes were the mean time taken to complete each entry, the number of completed data points, and quantitative feedback scores. As the highest level of clustering, participant number was entered as the random effect.

Bootstrapping was used to account for the use of non-normal distributed variables in all analysis. This has been suggested as an appropriate alternative when parametric assumptions are not met [21]. The variables were manually standardized in order to aid the interpretation of the results.

## Results

We were not able to keep a systematic record of how many individuals were approached by their care coordinators for participation and declined. However, it was the impression of clinical teams that the refusal rate was around 30%. Of the 38 individuals who were referred to the research team, 8 changed their mind, 3 were unable to be contacted, and 3 were deemed ineligible prior to consent. This provided a final sample of 24 individuals, all of whom completed the feedback assessments (ie, no participants withdrew from the study). One individual, however, did ask for the SMS text-only assessment to be ended 2 days early because she found it was making her ruminative.

### Testing for an Interaction Between Sampling Period and Method of Assessment

First, we assessed whether there was an interaction between the sampling period and device type. Regression analysis showed that the order of the two conditions did not significantly predict the total number of entries an individual completed (β =.06, SE.22, *P*=0.78), nor the length of time it took to complete each entry (β = -.06, SE.20, *P*=.78). However, starting with the SMS text-only implementation did show nonsignificantly increased negative appraisals of the devices (β = -.35, SE.20, *P*=.08).

### Comparing Native Smartphone Application and SMS Text-only Implementation

The number of entries completed during sampling was strongly correlated between the two delivery modalities (rho = 0.61, *P*=.002). When controlling for order effect, participants completed significantly greater numbers of entries on the native smartphone application when compared to SMS text-only implementation (Tables 3 and 4). Past studies have defined compliance as completing at least one-third of all possible data -points [22]. Using this criterion, 88% (n=21) of individuals were compliant when using the native smartphone application, whereas 71% (n=19) were compliant when using SMS text-only implementation. Participants completed a significantly greater number of entries in week one (mean = 16.4, SD 16.4) when compared to week two of sampling (mean = 13.7, SD 6.5; β = -.22, SE=.11, *P*=.04). When broken down by day, participants completed a mean of 3.4 (SD 0.8) entries on day 1, 3.8 (SD 0.5) on day 2, 3.0 (SD 1.2) on day 3, 3.4 (SD 1.1) on day 4, 3.0 (SD 1.3) on day 5, and 3.1 (SD 1.2) entries on day 6 when using the smartphone application. When using the SMS system this was lower at 2.3 (SD 1.4) entries on day 1, 2.4 (SD 1.3) entries on day 2, 2.6 (SD 1.5) entries on day 3, 2.6 (SD 1.3) entries on day 4, 2.0 (SD 1.4) entries on day 5, and 1.9 (SD 1.7) entries on day 6.

The length of time taken to complete each entry was highly correlated between the delivery modalities (rho = 0.62, *P*=.02), suggesting that those individuals who took longer on the native smartphone application also took longer to complete the SMS text-only implementation. However, as can be seen in Tables 3 and 4, individuals took an average 68.4 seconds (SD 39.5) to complete a full set of questions on the native smartphone application and 325.5 seconds (SD 145.6) on the SMS text-only implementation (β = 0.78, SE 0.09, *P*<.001). Thus, questions sets took 4.8 times longer on the SMS text-only implementation, than on native smartphone application. There was no significant difference in the length of time (seconds) it took participants to complete entries in week 1 (mean = 210.4, SD 171.3), when compared to week 2 (mean = 183.5, SD 166.3; β = -.08, SE=.09, *P*=.36) of sampling.

The total quantitative feedback scores for each device were also strongly correlated (rho = 0.48, *P*=0.02), suggesting that appraisals of the procedure were similar across both delivery modalities. No significant difference was observed for the total quantitative feedback score when comparing the two delivery modalities. Although none of the individual quantitative feedback items were significantly different, nonsignificantly higher scores (suggesting greater agreement) were observed for SMS text-only implementation on the items “Were there times where you had to stop doing something in order to answer the questions?”, ”Were there times when you felt like not answering?”, and “Was filling in the questions inconvenient?” (Table 3).

When considering an order effect, participants scored higher on the items “Were there times when you felt like not answering?” (week 1 mean 2.2, SD 1.1), week two mean 3.1, SD 2.2; β = 0.26, SE=.12, *P*=.03) and “Was it difficult to keep the device with you or carry it around?” (week 1 mean 1.8, SD 1.2; week 2 mean 2.6, SD 1.9; β = 0.26, SE=.12, *P*=.03) in the second, when compared to the first week of sampling. No other significant order effects were observed on the quantitative feedback scores.

Participants also reported the maximum length of time within which they would hypothetically be willing to complete questions on each delivery modality, which is displayed in Table 5. At the end of the final period of sampling, 67% (n=16) of individuals preferred the native smartphone application, 13% (n=3) of people preferred SMS text-only implementation, and 21% (n=5) of people had no preference. Additionally, 71% (n=17) of individuals found the native smartphone application easier to use, 17% (n=4) of people found SMS text-only implementation easier to use, and 13% (n=3) of people had no preference. Worthy of note is that 2 out of the 3 individuals who preferred their own phone currently owned a smartphone, which they used to complete the SMS text-only implementation.

**Table 3:** Quantitative feedback scores for the native smartphone application and SMS text-only implementation, and summary statistics.

	Native smartphone application				SMS text-only implementation					
	Mean	SD	Min	Max	Mean	SD	Min	Max	β^*P*^	SE
										
Time taken to complete questions (seconds)	68.4	39.5	18.8	179.7	325.5	145.6	118.8	686.9	0.78^<.001^	0.09
Number of entries completed	16.5	5.5	4.0	24.0	13.5	6.6	0.0	24.0	-0.25^.02^	0.11
Did answering the questions take a lot of work?	1.8	1.1	1	5	2.3	1.6	1	6	0.17	0.13
Were there times when you felt like not answering?	2.3	1.3	1	5	3.0	2.1	1	7	0.21^.07^	0.12
Did answering the questions take up a lot of time?	1.7	0.9	1	4	2.3	1.6	1	7	0.24	0.14
Were there times where you had to stop doing something in order to answer the questions?	3.4	1.7	1	7	4.1	1.7	1	7	0.20^.10^	0.12
Was it difficult to keep track of what the questions were asking you?	1.6	1.2	1	7	1.9	1.7	1	7	0.11	0.15
Were you familiar with using this type of technology?	4.7	2.3	1	7	5.3	2.2	1	7	0.14	0.14
Was it difficult to keep the device with you or carry it around?	1.9	1.4	1	6	2.4	1.8	1	6	0.16	0.12
Did you ever lose or forget the device?	1.7	0.9	1	4	1.8	1.4	1	6	0.06	0.13
Was using the key pad/touch screen difficult to use?	2.0	1.3	1	5	1.8	1.4	1	6	-0.08	0.15
Do you think other people would find the software easy to use?	5.3	1.8	2	7	5.9	1.4	3	7	0.19	0.16
Do you think you could make use of this approach in your everyday life?	4.0	1.8	1	7	3.9	2.2	1	7	-0.02	0.13
Do you think that this approach could help you or other service users?	5.3	1.9	1	7	5.6	1.2	3	7	0.11	0.15
Overall, this experience was stressful.	1.8	1.1	1	5	1.8	1.3	1	6	-0.04	0.18
Overall, this experience was challenging.	2.2	1.6	1	7	2.7	1.7	1	6	0.16	0.13
Overall, this experience was pleasing.	3.7	2.0	1	7	3.7	1.7	1	7	0.01	0.11
Did filling in the questions make you feel worse?	1.8	1.1	1	5	2.1	1.4	1	5	0.14	0.15
Did filling in the questions make you feel better?	2.8	1.5	1	6	3.0	1.6	1	7	0.08	0.13
Did you find the questions intrusive?	2.2	1.2	1	4	2.6	1.8	1	7	0.15	0.14
Was filling in the questions inconvenient?	2.0	1.0	1	4	2.5	1.4	1	5	0.23^.09^	0.14
Did you enjoy filling in the questions?	3.6	2.0	1	7	3.7	1.6	1	7	0.01	0.12
Total quantitative feedback score (positive items reversed):	53.0	11.2	33	76	56.2	14.2	27	88	0.13	0.10

**Table 4:** Quantitative feedback scores for the native smartphone application and SMS text-only implementation–momentary assessment symptom scores.

Momentary assessment symptom scores	Native smartphone application				SMS text-only implementation			
	Mean	SD	Min	Max	Mean	SD	Min	Max
Hallucinations	2.7	1.8	1.0	6.5	2.5	1.8	1.0	6.0
Anxiety	2.8	2.0	1.0	6.3	2.1	1.4	1.0	6.4
Grandiosity	2.3	1.5	1.0	6.0	2.3	1.4	1.0	6.0
Delusions	2.0	1.3	1.0	5.1	1.9	1.1	1.0	4.7
Paranoia	2.9	1.8	1.1	6.5	2.6	1.7	1.0	7.0
Hopelessness	3.2	1.4	1.0	5.7	3.0	1.3	1.0	5.1

**Table 5:** Maximum length of time willing to complete questions on the two implementations.

Maximum length of time willing to complete questions	Smartphone	Text messages
	n (%)	n (%)
U<2 weeks	2 (8%)	3 (13%)
2-3 weeks	10 (42%)	10 (42%)
3-4 weeks	1 (4%)	5 (21%)
4-5 weeks	3 (13%)	1 (4%)
5+ weeks	8 (33%)	5 (21%)

## Discussion

The objective of this study was to compare two different delivery modalities of the same diagnostic assessment for individuals with nonaffective psychosis—a native smartphone application employing a graphical, touch user interface against an SMS text-only implementation. The overall hypothesis of the study was that participants with serious mental illness would find both systems feasible and acceptable to use, measured by the quantitative postassessment feedback questionnaire scores, the number of data points completed, and the time taken to complete the assessment. We also predicted that participants would complete a significantly greater number of data points, in less time, when using a native smartphone application when compared to the text messages and that the former software would be more positively appraised. The length of time that participants would hypothetically be willing to complete questions was also assessed in order to gauge the feasibility of longer-term assessment.

In line with the hypotheses, participants completed a significantly greater number of entries on the native smartphone application (69%, mean = 16.5), when compared to the text message interface (56%, mean = 13.5). It is desirable to keep missing data to a minimum in order to generate a representative picture of an individual’s symptoms over time. Other studies have observed similarly high rates of compliance to the native smartphone application when using PDAs (ie, 69-72%) [7,23]. The increased usability and streamlined, graphical interface seen in purpose built software applications may encourage greater levels of compliance than those of SMS text-only implementations.

On average, sets of questions on the SMS text-only implementation took 4.8 times longer to complete when compared to the native smartphone application, which may have contributed to the reduced rates of compliance seen with this method of sampling. Although just failing to reach statistical significance, the quantitative feedback highlighted greater disruption to activities and inconvenience and less inclination to complete the questions when participants used the SMS text-only implementation. This is perhaps understandable given the greater time investment incurred by this method. Past research has suggested that the movement of mobile technology from the foreground to the background of an individual’s life represents an important step in the accommodation of these technologies [24], which may be less likely if it perceived as burdensome.

Somewhat surprisingly, the two delivery modalities did not differ in any of the other quantitative feedback items or the total quantitative feedback score. The mean scores suggest that these appraisals were generally positive across both conditions. Thus, both forms of technology were deemed acceptable and well integrated and may represent suitable methods for facilitating real-time assessment. However, in the forced choice questions, the majority of the sample stated that they preferred the native smartphone application and that they found it easier to use, suggesting that this may be a more attractive delivery modality. Of the three individuals who preferred the SMS text-only implementation, two currently owned a smartphone, which they used for completing the text messages. These mobile phones may have allowed for a more streamlined text system (eg, threaded messages) reducing the differences between the two types of devices.

While the mean quantitative feedback scores suggested generally positive appraisals of both delivery modalities, these varied considerably between individuals. For example, some participants stated that they felt this technology could help them, whereas others were more skeptical about its advantages. Some participants reported mild negative reactivity to the method. It is possible that mobile phone assessment is less suitable in certain subgroups of patients, and in its future application, reactivity should be carefully monitored.

As this technology makes the transition from research to real-world clinical application, it will be vital to assess the feasibility and uptake of this software over longer periods of time and the factors influencing nonparticipation and withdrawal. In this study, participants completed fewer entries in the second week of sampling. Additionally, only a third of participants said they would be willing to complete the procedure for 5 or more weeks with the native smartphone application, with an even lower percentage for the SMS text-only implementation (21%). In the future, it may be necessary to employ machine learning in order to tailor the choice of questions and sampling rates to the service user [25], while placing particular emphasis on symptoms of primary concern to their clinical team. Person-tailored sampling could increase the feasibility of conducting longer-term real-time assessment. Automated and clinician-delivered feedback, or monetary incentives, may also promote acceptance and compliance.

In conclusion, this study provides data to suggest that both native smartphone applications and SMS text-only implementation represent acceptable technologies for facilitating real-time assessment in individuals with nonaffective psychosis. However, the native smartphone application was found to be preferable to SMS text-only implementation in terms of greater data point completion and shorter response times. Limitations of this study include the relatively modest length of the sampling procedure and moderate rates of nonparticipation by those approached to take part. In the future, it will be important to upload software applications onto individual’s own phone rather than issuing them with an additional device.

## Abbreviations

PANSS: Positive and Negative Syndrome Scale.
PDA: personal digital assistant
SMS: short message service
